# Subclinical alterations in left ventricular structure and function according to obesity and metabolic health status

**DOI:** 10.1371/journal.pone.0222118

**Published:** 2019-09-12

**Authors:** Hyun-Jung Lee, Hack-Lyoung Kim, Woo-Hyun Lim, Jae-Bin Seo, Sang-Hyun Kim, Joo-Hee Zo, Myung-A. Kim

**Affiliations:** Division of Cardiology, Department of Internal Medicine, Seoul National University College of Medicine, Seoul National University Boramae Medical Center, Seoul, Korea; Universita degli Studi di Roma La Sapienza, ITALY

## Abstract

**Background:**

Obesity and metabolic syndrome (MetS) are associated with high risk of cardiac dysfunction and heart failure. We assessed the effect of obesity and metabolic health status on left ventricular (LV) structure and function in subjects without overt heart disease.

**Methods:**

In 789 subjects (58.8±13.0 years, 50.7% males) without overt heart disease, LV morphology and function were compared among 6 groups stratified by body mass index (BMI) (normal weight, overweight and obese) and metabolic health status (meeting ≤1 criterion of MetS excluding waist circumference defined as metabolically healthy; otherwise, metabolically unhealthy).

**Results:**

LV ejection fraction (LVEF) was not different among the 6 groups (P>0.05). However, high BMI and poor metabolic health were associated with poorer global longitudinal strain (GLS), higher LV mass index (LVMI) and higher E/e′ (P<0.001). Poor metabolic health status was associated with greater adverse changes in LV structure and function than obesity, and among MetS components, high systolic blood pressure (SBP) showed the greatest impact. Higher SBP, BMI and triglycerides were independently associated with worse GLS, and higher SBP was also associated with worse LVMI and E/e´. GLS, LVMI and E/e´ worsened in proportion to the number of MetS criteria or continuous MetS scores. Adverse myocardial changes associated with obesity were significant in the metabolically healthy group, but not in the metabolically unhealthy group.

**Conclusions:**

Obesity and poor metabolic health status were associated with subclinical decrement in LV systolic and diastolic function, and higher LV mass, but not with LVEF, in subjects without overt heart disease.

## Introduction

Obesity[1–3] and metabolic syndrome (MetS), as well as the individual components of MetS,[4–6] are associated with high risk of heart failure and cardiovascular diseases. Even in subjects without overt heart disease, obesity or MetS can cause adverse changes in cardiac structure and function, which may precede the development of heart failure. Advances in echocardiographic imaging techniques have made it possible to assess subclinical cardiac dysfunction. Notably, left ventricular (LV) global longitudinal strain (GLS) by speckle-tracking echocardiography can detect subtle systolic dysfunction before changes in LV ejection fraction (LVEF) and predict development of heart failure in asymptomatic individuals.[7–9] Obesity has been reported to be associated with LV dilatation, LV hypertrophy, LV diastolic dysfunction as well as worse LV strain, though LVEF is typically preserved or only mildly impaired.[10–13] MetS has also been associated with LV hypertrophy, LV diastolic dysfunction and subtle LV systolic dysfunction with preserved LVEF.[14–17]

We aimed to assess the relationship of obesity and metabolic health status with LV structure and function in adults without overt heart disease and to examine the differences in myocardial function depending on different obesity and metabolic phenotypes. We also examined the degree to which obesity or MetS and its components contribute to various aspects of myocardial function, especially subclinical LV systolic dysfunction represented by GLS.

## Methods

### Study population

This retrospective single-center study included consecutive subjects (age ≥ 18 years) without overt heart disease who underwent transthoracic echocardiography including measurement of GLS from March 2012 to June 2016. There were 2,788 individuals with GLS measurements, and the following subjects were excluded thereof if they had (i) LVEF < 50% (n = 169), (ii) regional wall motion abnormalities (n = 77), (iii) LV hypertrophy (n = 9), (iv) significant coronary artery disease (myocardial infarction or revascularization) (n = 399), (v) symptomatic heart failure (n = 22), (vi) valvular dysfunction more than mild degree (n = 18), (vii) stress-induced cardiomyopathy (n = 2), (viii) hypertrophic cardiomyopathy (n = 4), (ix) infiltrative cardiomyopathy (n = 3), (x) congenital heart disease (n = 4), xi) pericardial effusion (n = 7), (xii) significant arrhythmia (atrial fibrillation, supraventricular tachycardia, left bundle branch block, very frequent ventricular premature contractions or ventricular tachycardia, etc.) (n = 47), (xiii) pulmonary thromboembolism (n = 5), xiv) recent stroke (n = 171), (xv) chemotherapy for cancer (n = 131), (xvi) end-stage renal disease (n = 22), (xvii) liver cirrhosis (n = 5), (xviii) systemic inflammation (n = 25), or (xix) shock (n = 3). Of the 1,485 remaining subjects, subjects without data on body mass index (BMI) (n = 339) or MetS criteria (blood pressure, fasting glucose or glycated hemoglobin [HbA1c] levels, high-density lipoprotein [HDL] cholesterol and triglyceride levels) (n = 317) were excluded. Also, underweight subjects with BMI < 18.5 kg/m^2^ were excluded (n = 40). After these exclusions, a total of 789 adults were finally analyzed in the present study (**S1 Fig**). The study protocol was approved by the Institutional Review Board (IRB) of Boramae Medical Center (Seoul, Korea) (IRB No. 10-2018-3), and informed consent was waived due to the retrospective study design and routine nature of information collected.

Subjects were classified by BMI as normal weight (18.5–22.9 kg/m^2^), overweight (23–24.9 kg/m^2^) and obese (≥ 25 kg/m^2^), according to World Health Organization (WHO) Asia-Pacific definitions of obesity and BMI groups.[18] Being ‘metabolically healthy’ was defined as meeting ≤1 criterion of MetS excluding waist circumference:[19] (i) elevated blood pressure (systolic blood pressure [SBP] ≥ 130 mm Hg or diastolic blood pressure [DBP] ≥ 85 mm Hg) or use of relevant medications, (ii) elevated fasting glucose (≥100 mg/dL) or HbA1c ≥ 5.7%, or use of relevant medications,[20] (iii) elevated triglycerides (≥150 mg/dL) or use of relevant medication, and (iv) low HDL cholesterol (<40 mg/dL for men and <50 mg/dL for women). Participants were stratified by BMI categories and metabolic health status as metabolically healthy normal weight (MHNW), metabolically healthy overweight (MHOW), metabolically healthy obese (MHO), metabolically unhealthy normal weight (MUNW), metabolically unhealthy overweight (MUOW), or metabolically unhealthy obese (MUO). We also performed analyses defining ‘metabolically healthy’ as having no MetS criteria.

### Transthoracic echocardiography

All subjects underwent transthoracic echocardiography, including tissue Doppler imaging and speckle tracking (GE Medical Systems, Milwaukee, WI, USA) using a 2.5-MHz probe. Measurements were made in accordance with the latest guidelines.[21–23] LV chamber dimensions and wall thickness were measured by M-mode, relative wall thickness (RWT) was calculated as 2-fold posterior wall thickness divided by the LV end-diastolic dimension. LV mass index (LVMI) was calculated with a validated formula and indexed to the body surface area. Left atrial volume was measured using the biplane modified Simpson method and was indexed to the body surface area (left atrial volume index, LAVI). LVEF was also calculated using Simpson’s biplane method. Doppler was used to measure mitral E and A wave velocities, deceleration time; tissue Doppler was used to measure mitral septal annular early (e´) and late (a´) diastolic velocities. E/e´ was calculated as an index of LV-filling pressure. Speckle-tracking analysis was performed semi-automatically using a commercially available software (GE Medical Systems) during a routine echocardiographic examination, with manual adjustment by a sonographer. GLS was the average of negative peak longitudinal strain from 17 ventricular segments obtained from the apical 4-chamber, 3-chamber and 2-chamber views. All measurements were averaged through 3 consecutive cardiac cycles. Two experienced sonographers performed the echocardiography. The coefficient of variation for GLS measurements was 2.09%, showing excellent intra-observer reproducibility. The correlation coefficient for inter-observer agreements was 0.92 for E/e′ in our laboratory.

### Data collection

Electronic medical records were reviewed for clinical information, laboratory data and medication at the time of echocardiography. BMI was calculated as weight in kilograms divided by the square of height in meters (kg/m^2^). SBP, DBP and heart rate were measured by a trained nurse using an oscillometric device; subjects on antihypertensive medication were given notice to take them as usual. Subjects were classified as having hypertension, diabetes mellitus or dyslipidemia if they had been diagnosed by a physician and were on therapy. Smoking status was assessed by interview, and subjects were classified as current smokers if they had smoked regularly during the previous 12 months. Venous sampling was done after an overnight fast for fasting glucose, total cholesterol, low-density lipoprotein (LDL) cholesterol, HDL cholesterol, triglyceride, C-reactive protein (CRP) and creatinine levels. Estimated glomerular filtration rate (GFR) was calculated using 4-component Modification of Diet in Renal Disease (MDRD) Study equation incorporating age, race, sex and serum creatinine level.[24] The MetS score is a continuous score calculated as the sum of age- and sex-standardized absolute Z-scores (standardized residuals) for mean arterial blood pressure, glucose, HDL cholesterol, triglycerides and BMI,[25, 26] which has mostly been used in children or adolescents due to a lack of uniform definition for pediatric metabolic abnormalities. As there are more homogeneous cut-offs for metabolic abnormalities in adults, the use of MetS score is not common in the adult population. However, while the definition of MetS is only binary, the MetS score can be more useful as a continuous scale representing composite cardiovascular disease risk factor profile. Indeed, the usefulness of MetS score in adult has also been demonstrated, and several recent studies have been conducted using the MetS score in adults[27, 28].Thus, we calculated the MetS scores for the study population, to represent degree of metabolic unhealthiness.

### Statistical analysis

Data are presented as numbers and percentages for categorical variables, and as means with standard deviations for continuous variables. Univariable statistical comparisons of patient characteristics and echocardiography parameters were conducted: to compare categorical variables, the chi-square test; to compare continuous variables between 2 groups, the Student’s t-test; to compare continuous variables among 3 or more groups, the analysis of variance (ANOVA) or the Welch test if homogeneity of variances was not met, and *post hoc* analysis with the Bonferroni method for the former and the Dunnett T3 method for the latter. P-values for trend among 3 or more groups were analyzed using the Jonckheere-Terpstra test for continuous variables and linear-by-linear analysis for categorical variables. P-values for trend were analyzed in the order of MHNW, MHOW, MHO, MUNW, MUOW and MUO; also, linear regression analyses were done with groups as a continuous variable in the order of MHNW (0, reference), MHOW (1), MHO (2), MUNW (3), MUOW (4), and MUO (5). Whether the impact of metabolic phenotype groups differed according to sex and whether there was an interaction with sex were assessed with two-way ANOVA. Of echocardiography parameters, LVEF, GLS, E/e´ and LVMI values were adjusted for sex and age, and compared among the groups using analysis of covariance (ANCOVA) and Bonferroni *post hoc* analysis.

Associations between MetS and obesity parameters, and GLS, E/e´ and LVMI were assessed by linear regression analysis, with calculation of β estimates representing the change in GLS (%), LVMI (g/m^2^) or E/e’ per 1-SD change in continuous clinical traits. The degree of metabolic unhealthiness was represented by the number of MetS criteria or continuous MetS scores. Correlations between MetS scores and GLS, E/e´ and LVMI were shown with scatter plots and fitted lines with Pearson’s correlation coefficient (*r*). Univariable unadjusted analysis was done using simple linear regression, and multivariable adjusted analysis using multiple linear regression. Multivariable regression analyses were adjusted for age, sex, BMI, SBP, glucose, triglycerides, HDL-C and other significant covariates on univariable analysis, except in the case of MetS criteria and MetS score, which were only adjusted for age and sex.

Two-sided *P* values <0.05 were considered statistically significant. For Bonferroni *post hoc* analyses, *P* values were adjusted for the number of comparisons by SPSS (raw *post hoc* p-value multiplied by the number of comparisons) so that <0.05 could be considered significant. All statistical analyses were performed using SPSS version 25 (IBM Co., Armonk, NY, USA).

## Results

### Clinical characteristics of the study population

The mean age of the study subjects was 58.8 ± 13.0 years, and 50.7% were male. The majority of subjects (67.9%) were metabolically unhealthy. The proportions of normal-weight, overweight and obese subjects were 31.8%, 26.1% and 42.1%, respectively. The study population was further divided into 6 groups according to metabolic health status (metabolically healthy vs. unhealthy) and obesity (normal weight vs. overweight vs. obese): MHNW (n = 111, 14.1%), MHOW (n = 76, 9.6%), MHO (n = 66, 8.4%), MUNW (n = 140, 17.7%), MUOW (n = 130, 16.5%), and MUO (n = 266, 33.7%). Comparisons of clinical characteristics among these 6 groups are shown in **Table 1** and **S1 Table**. Metabolically unhealthy subjects were older, and had greater BMI, elevated blood pressure, more cardiovascular risk factors and unfavorable laboratory results such as high glucose level, more atherogenic lipid profiles and lower GFR than metabolically healthy ones. Cardiovascular medications were more frequently prescribed in the metabolically unhealthy group than in the metabolically healthy group (S1 Table). Similarly, as BMI increased from normal to obese, there were trends toward higher blood pressure, higher prevalence of cardiovascular risk factors, higher levels of HbA1c and triglycerides, and lower levels of HDL cholesterol and GFR. Cardiovascular medications were most frequently prescribed to the obese group, followed by the overweight and normal group (S1 Table). In general, high prevalence of cardiovascular risk factors and metabolic derangement was more strongly associated with poor metabolic health status than with obesity.

**Table 1:** Comparison of clinical characteristics among metabolic phenotypes.

Characteristic	Total (n = 789)	MHNW (n = 111)	MHOW (n = 76)	MHO (n = 66)	MUNW (n = 140)	MUOW (n = 130)	MUO (n = 266)	*P*
Age, years	58.8±13.0	53.8±13.9	53.7±11.7	53.2±15.0	64.3±12.1	61.7±10.2	59.6±12.4	**< 0.001**
Male sex, n (%)	400 (50.7)	49 (44.1)	34 (44.7)	33 (50.0)	72 (51.4)	70 (53.8)	142 (53.4)	0.505
Body mass index, kg/m^2^	24.6±2.9	21.4±1.2	24.0±0.6	27.0±2.1	21.5±1.1	24.1±0.6	27.4±2.0	**< 0.001**
Systolic blood pressure, mmHg	130±17	121±15	120±13	123±10	134±18	131±18	136±17	**< 0.001**
Diastolic blood pressure, mmHg	78.7±11.6	73±12	75±10	76±8	81±11	79±11	82±12	**< 0.001**
Heart rate, beats/min	67.6±11.1	64.2±8.7	68.3±10.9	65.0±10.2	69.9±12.0	66.4±12.1	68.9±10.9	**0.014**
Risk factors, n (%)								
Hypertension	408 (51.7)	17 (15.3)	10 (13.2)	12 (18.2)	92 (65.7)	81 (62.3)	196 (73.7)	**< 0.001**
Diabetes mellitus	141 (17.9)	3 (2.7)	3 (3.9)	4 (6.1)	36 (25.7)	26 (20.0)	69 (25.9)	**< 0.001**
Dyslipidemia	171 (21.7)	6 (5.4)	2 (2.6)	2 (3.0)	36 (25.7)	40 (30.8)	85 (37.6)	**< 0.001**
Current smoker	144 (18.3)	20 (18.0)	14 (18.4)	14 (21.2)	23 (16.4)	25 (19.2)	48 (18.0)	0.977
Major laboratory results								
Fasting blood glucose, mg/dL	113±53	98±17	98±15	99±15	122 ±63	117±32	121±72	**< 0.001**
Hemoglobin A1c, %	6.0±1.0	5.5±0.4	5.5±0.4	5.6±0.5	6.2±1.1	6.1±0.9	6.3±1.2	**< 0.001**
Total cholesterol, mg/dL	182±40	184±33	187±31	182±33	180±48	181±41	180±41	0.847
LDL cholesterol, mg/dL	114±35	113±30	116±28	113±30	115±41	112±35	113±36	0.974
HDL cholesterol, mg/dL	49.8±12.8	58.4±13.4	56.3±9.8	54.1±12.6	48.7±13.3	47.2±11.4	45.2±10.7	**< 0.001**
Triglyceride, mg/dL	124±89	86±33	97±35	96±28	111±57	140±84	152±125	**< 0.001**
C-reactive protein, mg/dL	0.40±1.31	0.32±1.49	0.34±1.05	0.39±0.87	0.57±1.97	0.26±0.68	0.44±1.20	0.660
GFR, mL/min/1.73m^2^	89.3±22.6	96.7±18.5	90.9±20.0	93.5±27.8	88.3±22.5	84.0±18.1	87.9±24.6	**< 0.001**

Groups were compared with the chi-square test and analysis of variance (ANOVA) or Welch test, and bold values indicate significant differences of *P* < 0.05

MHNW, metabolically healthy normal weight; MHOW, metabolically healthy overweight; MHO, metabolically healthy obese; MUNW, metabolically unhealthy normal weight; MUOW, metabolically unhealthy overweight; MUO, metabolically unhealthy obese; LDL, low-density lipoprotein; HDL, high-density lipoprotein; GFR, glomerular filtration rate.

### Comparisons of echocardiographic parameters

Comparisons of echocardiographic parameters among 6 groups are demonstrated in **Table 2** (by ANOVA and Bonferroni *post hoc* analysis, or if unequal variances, by Welch test with Dunett T3 *post hoc* analysis). Significant p-values of *post hoc* analyses are shown in **S2 Table**. As shown in **Table 2**, there were no significant differences in LVEF among the 6 groups (*P* > 0.05 for each). However, GLS worsened if subjects were more obese or metabolically unhealthy, in the order of MHNW, MHOW, MHO, MUNW, MUOW and MUO (worst) (*P* for trend < 0.001). LVMI and RWT were significantly different among the 6 groups, with MHNW having the lowest values and MUOW having the highest values (*P* < 0.001). Metabolically unhealthy groups showed higher LVMI and RWT than MHNW and MHOW groups, and the MHO group showed intermediate values. Parameters related to LV diastolic function, including A wave velocity, E/A, e´, E/e´ and LAVI, were significantly different among the 6 groups, with MHNW having the most favorable values (*P* < 0.001).

**Table 2:** Comparison of echocardiography parameters among metabolic phenotypes.

Echocardiography parameters	Total (n = 789)	MHNW (n = 111)	MHOW (n = 76)	MHO (n = 66)	MUNW (n = 140)	MUOW (n = 130)	MUO (n = 266)	*P*	*P for trend*
LVEF, %	66.7±5.1	66.8±4.5	66.3±4.5	66.7±4.2	66.9±6.6	66.5±5.7	66.9±4.4	0.935	0.728
GLS, %	-18.90±2.50	-19.91±2.29^a,b,c^	-19.53±2.20^d^	-19.05±2.19	-18.84±2.80^a^	-18.50±2.55^b^	-18.48±2.40^c,d^	**< 0.001**	**<0.001**
LVMI, g/m^2^	85.3±18.4	78.1±15.2^a,b,c^	78.1±15.3^d,e,f^	82.6±18.3^g^	88.9±19.6^a,d^	91.4±20.3^b,e,g^	86.0±17.3^c,f^	**< 0.001**	**< 0.001**
RWT	0.36±0.05	0.34±0.04^a,b,c,d^	0.34±0.04^e,f,g^	0.36±0.05^a^	0.36±0.05^b,e^	0.38±0.04^c,f^	0.37±0.05^d,g^	**< 0.001**	**< 0.001**
E, m/s	0.66±0.24	0.68±0.16	0.66±0.16	0.66±0.19	0.66±0.16	0.64±0.14	0.66±0.35	0.908	0.364
A, m/s	0.75±0.37	0.63±0.16^a,b,c^	0.65±0.17^d^	0.70±0.21	0.78±0.19^a^	0.78±0.19^b^	0.81±0.58^c,d^	**< 0.001**	**< 0.001**
E/A	0.94±0.34	1.15±0.42^a,b,c^	1.08±0.37^d,e,f^	1.02±0.42	0.88±0.29^a,d^	0.86±0.28^b,e^	0.86±0.28^c,f^	**< 0.001**	**< 0.001**
DT, ms	211±48	203±43	199±42	212±46	210±53	217±44	216±50	0.019	**0.001**
e′, cm/s	9.6±7.2^a,b,c^	8.0±2.6^d,e,f^	7.4±2.4	6.8±2.1^a,d^	6.8±2.0^b,e^	6.6±1.8^b,e^	9.6±7.2^a,b,c^	**< 0.001**	**< 0.001**
E/e′	9.64±3.18	7.97±2.14^a,b,c,d^	8.83±2.55^e,f^	9.46±3.29^a^	10.20±3.38^b,e^	9.97±3.07^c^	10.15±3.18^d,f^	**< 0.001**	**< 0.001**
LAVI, mL/m^2^	29.0±8.4	26.6±6.6^a,b,c^	27.6±6.6	27.3±6.3	30.4±9.5^a^	30.5±11.0^b^	29.3±7.6^c^	**< 0.001**	**< 0.001**
TR Vmax, m/s	2.2±0.3	2.2±0.3	2.2±0.3	2.2±0.2	2.3±0.3	2.2±0.3	2.3±0.2	0.101	**0.010**

Bold values indicate significant differences of *P* < 0.05 for comparison of groups: analysis of variance (ANOVA) with Bonferroni *post hoc* analysis for LVEF, GLS, LVMI, RWT, E, A, DT, TR Vmax; Welch test with Dunett T3 *post hoc* analysis for E/A, e’, E/e’, LAVI. Superscript letters indicate significant differences between the marked groups in *post hoc* analysis (significant p-values in *post hoc* analyses are available in S2 Table). P for trend was analyzed in the order of MHNW, MHOW, MHO, MUNW, MUOW and MUO.

MHNW, metabolically healthy normal weight; MHOW, metabolically healthy overweight; MHO, metabolically healthy obese; MUNW, metabolically unhealthy normal weight; MUOW, metabolically unhealthy overweight; MUO, metabolically unhealthy obese; LVEF, left ventricular ejection fraction; GLS, global longitudinal strain; LVMI, left ventricular mass index; RWT, relative wall thickness; DT, deceleration time; LAVI, left atrial volume index; TR, tricuspid regurgitation; Vmax, maximal velocity.

Overall, most echocardiography parameters worsened if subjects were more obese or metabolically unhealthy, in the order of MHNW, MHOW, MHO, MUNW, MUOW and MUO (worst) (*P* for trend < 0.001), with metabolic derangement being more detrimental than higher BMI (**Table 2**). Exceptions were LVEF and mitral E, which were not significantly different among the groups. Linear regression analysis for the association between metabolic phenotypes and echocardiography parameters with groups as an ordinal variable in the order of MHNW (0), MHOW (1), MHO (2), MUNW (3), MUOW (4), and MUO (5) also showed similar results (**S3 Table**); there was a significant association between 1-increase in group number and worsening of most echocardiography parameters, which persisted after adjustment for age and sex, with the exception of LVEF, E and TR Vmax.

There was significant sex differences in echocardiography parameters: men showed slightly worse LV systolic function (worse LVEF, GLS), higher LV mass (higher LVMI, RWT) and better diastolic function (higher E and lower A, E/e’, LAVI, TR Vmax) compared to women. However, the impact of metabolic phenotypes on echocardiography parameters was consistent in both sexes (**S4 Table**): P-values for interaction were not significant for all echocardiography parameters.

In this study, being ‘metabolically healthy’ was defined as meeting ≤1 criterion of MetS excluding waist circumference. The trend of echocardiography parameters according to metabolic phenotype groups were consistent when ‘metabolically healthy’ was defined as having no MetS criteria (**S5 Table**).

**Fig 1:**
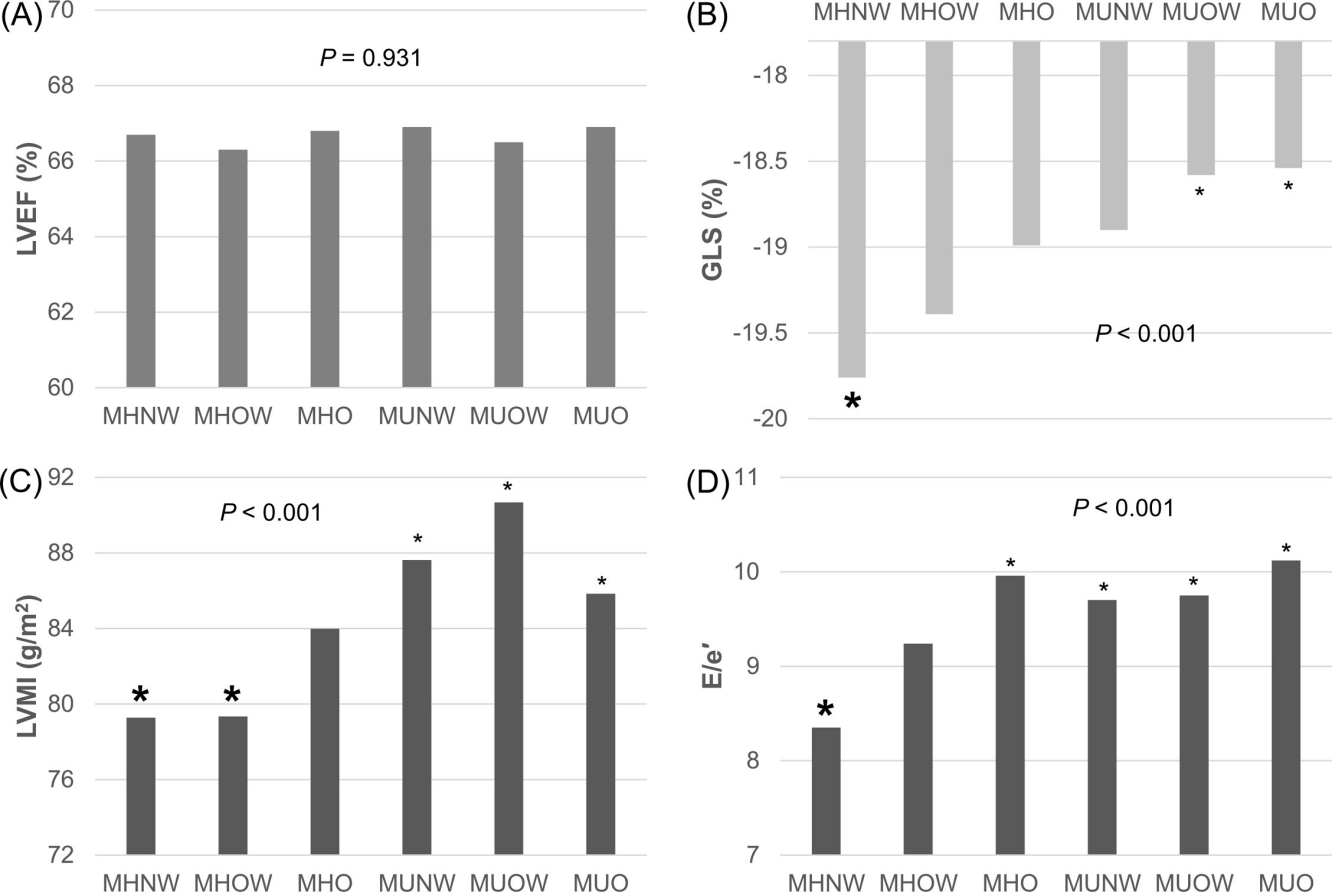
Comparisons of mean values of representative echocardiography parameters among groups, adjusted for age and sex. (A) Left ventricular ejection fraction (LVEF). (B) Global longitudinal strain (GLS). (C) Left ventricular mass index (LVMI). (D) E/e’. Groups noted with big asterisks showed significant differences from groups noted with small asterisks on *post hoc* analysis.MHNW, metabolically healthy normal weight; MHOW, metabolically overweight; MHO, metabolically healthy obese; MUNW, metabolically unhealthy normal weight; MUOW, metabolically overweight; MUO, metabolically unhealthy obese.

**Fig 1** demonstrates mean values of LVEF, GLS, LVMI and E/e´, adjusted for age and sex in the 6 groups. There was no difference in LVEF among the groups. Meanwhile, for GLS, LVMI and E/e’, MHNW showed the most favorable values. Generally, metabolically unhealthy groups showed the worst values, and MHO showed intermediate values.

As metabolic health status showed stronger associations with aggravated echocardiography parameters than obesity, we stratified the population by metabolic health status and compared the BMI categories within each stratum to assess the effect of BMI and obesity (**S6 Table**). In the metabolically healthy population, MHO was related with worse GLS, diastolic dysfunction (higher A, lower e´ and higher E/e´) and greater RWT (*P* < 0.05 for each). However, within the metabolically unhealthy population, there were no significant differences among the BMI categories except for LVMI.

### Clinical parameters associated with GLS, LVMI and E/e′

**Fig 2:**
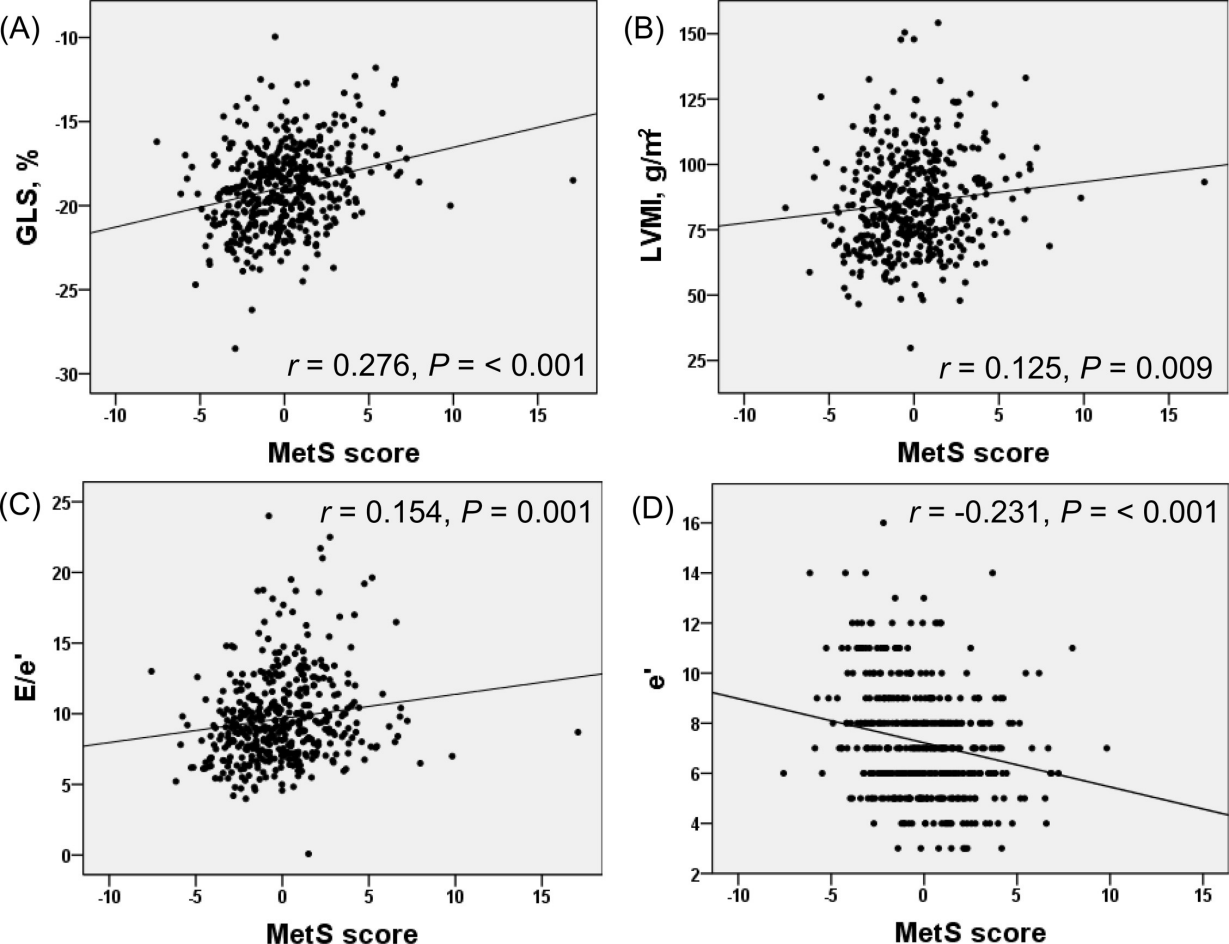
Scatter plots showing correlations between continuous MetS scores and representative echocardiography parameters. (A) Global longitudinal strain (GLS). (B) Left ventricular mass index (LVMI). (C) E/e’. (D) e’. *r* = Pearson’s correlation coefficient MetS, metabolic syndrome.

As poor metabolic health status was associated with aggravation of GLS, high LVMI and impaired diastolic function, we also assessed whether degree of poor metabolic health correlated with aggravation of these echocardiography parameters. The degree of poor metabolic health was represented by the number of MetS criteria and continuous MetS scores. **Fig 2** demonstrates correlations of MetS scores with GLS, LVMI, E/e′, and e′. GLS, LVMI, E/e′, and e′ all worsened in proportion to the number of MetS criteria and MetS scores, even after adjustment for age and sex (*P* ≤ 0.01 for each) (**Table 3**).

**Table 3:** Metabolic syndrome and obesity parameters associated with GLS, LVMI and E/e′.

Parameter	GLS				LVMI				E/e′			
	*Unadjusted*		Adjusted*		*Unadjusted*		Adjusted*		*Unadjusted*		Adjusted*	
	*β*	*P*	*β*	*P*	*β*	*P*	*β*	*P*	*β*	*P*	*β*	*P*
Summed variables												
Number of MetS criteria^†^^,^ ^‡^	0.205	**< 0.001**	0.178	**0.001**	0.161	**< 0.001**	0.107	**0.003**	0.245	**< 0.001**	0.172	**< 0.001**
MetS score^†^	0.276	**< 0.001**	0.264	**< 0.001**	0.125	**0.009**	0.120	**0.009**	0.154	**0.001**	0.161	**< 0.001**
Single variable												
BMI, per 1 kg/m^2^	0.152	**< 0.001**	0.128	**0.004**	0.010	0.783	-	-	0.077	**0.031**	0.059	0.251
SBP	0.271	**< 0.001**	0.226	**< 0.001**	0.236	**< 0.001**	0.180	**0.001**	0.275	**< 0.001**	0.257	**< 0.001**
Glucose	0.142	**< 0.001**	0.046	0.279	0.070	0.055	-	-	0.087	**0.017**	-0.080	0.937
LDL cholesterol	-0.042	0.280	-	-	-0.054	0.163	-	-	-0.109	**0.005**	-0.064	0.201
HDL cholesterol	-0.184	**< 0.001**	0.010	0.819	-0.137	**< 0.001**	-0.067	0.236	-0.080	**0.025**	-0.049	0.362
Triglycerides	0.117	**0.001**	0.094	**0.030**	0.026	0.467	-	-	-0.004	0.903	0.011	0.830

*β* estimate represents the change in GLS (%), LVMI (g/m^2^) or E/e’ per 1-SD change in continuous clinical traits.

*: adjusted for age, sex, BMI, SBP, glucose, triglycerides, HDL-C and other significant covariates on univariable analysis (except for †).

†: MetS criteria and MetS score were adjusted for age and sex.

‡: obesity substituted for waist circumference, in counting number of MetS criteria

GLS, global longitudinal strain; LVMI, Left ventricular mass index; MetS, metabolic syndrome; BMI, body mass index; SBP, systolic blood pressure; LDL, low-density lipoprotein; HDL, high-density lipoprotein.

Of metabolic and obesity factors, we assessed which showed the strongest associations with aggravation of GLS, LVMI and E/e′ (**Table 3**). Higher BMI, SBP and triglycerides were independently associated with worse GLS (*P* < 0.05 for each) in multivariable analysis. Of metabolic traits, SBP showed the strongest correlation with worse GLS (*β* = 0.226), followed by BMI (*β* = 0.128). Higher SBP was also independently associated with higher LVMI and E/e′ (*P* < 0.05 for each) in multivariable analysis.

## Discussion

We demonstrated that obesity and poor metabolic health were associated with poor GLS as well as high LVMI and E/e´, but not with LVEF in subjects without overt heart disease. Poor metabolic health status was related to more adverse changes in LV structure and function than obesity. Increased number of risk factors meeting MetS criteria or increased continuous MetS scores were associated with further worsening of GLS, LVMI and diastolic function. Among MetS components, high SBP showed the strongest association with poor GLS as well as high LVMI and E/e´. Obesity was also associated with worse GLS. Adverse myocardial changes associated with obesity were significant in the metabolically healthy group, but not in the metabolically unhealthy group.

Obesity and MetS in subjects without overt heart disease are known to be associated with adverse changes in LV structure and function, but the degree to which obesity or MetS and its components contribute to various aspects of myocardial function is not yet fully established. Obesity and MetS are associated with impaired LV systolic mechanics, though LVEF mainly remains preserved.[10–12, 14, 16] Subtle systolic dysfunction detected by GLS before impairment of LVEF can predict development of heart failure in asymptomatic individuals,[7–9] and many studies have shown the superiority of GLS over LVEF as a prognostic factor, especially in patients with mildly depressed or normal-range LVEF.[29–32] In a previous study (n = 190), while obesity *per se* was associated with impaired longitudinal strain and dyssynchrony in the absence of impairment in LVEF, there was no significant difference in GLS between MUO and MHO subjects,[33] suggesting that obesity may be of greater importance in impaired systolic mechanics. On the other hand, in another study (n = 67),[34] the magnitude of subclinical systolic and diastolic dysfunction correlated with the number of MetS traits, but not with BMI or degree of ectopic fat deposition. In this study, we found that while higher BMI and poor metabolic health status were both associated with impaired GLS, the latter was more strongly associated with worse GLS values. The magnitude of systolic dysfunction increased in proportion to the number of risk factors meeting MetS criteria or continuous MetS scores, which is consistent with the results of prior studies.[34, 35] We found that higher SBP, BMI and triglycerides among metabolic factors were significantly associated with worse GLS in multivariable analysis and that elevated SBP, showed the strongest association, followed by BMI. These results are in line with those of a previous study showing that higher SBP and waist circumference were associated with worse GLS,[33] and another study showing that BMI, central adiposity, insulin resistance and hypertriglyceridemia were associated with worse GLS (blood pressure was not evaluated in that study).[35]

Obesity and MetS are also associated with LV diastolic dysfunction.[11, 13, 15] We found that indices of diastolic function were impaired in both obese and metabolically unhealthy subjects, and poor metabolic health status was more strongly associated with impaired diastolic function than high BMI, which is consistent with results of previous studies.[33, 34, 36] Among metabolic factors, SBP was found to show the strongest association with E/e´.

Obesity and MetS are associated with LV hypertrophy as well.[10–12, 14, 16] A previous study showed that obesity is associated with high LVMI, regardless of the presence of MetS, but only those with MetS had high RWT.[33] We found that poor metabolic health status was associated with high LVMI and RWT, while BMI showed less clear associations. This suggests that poor metabolic health status is more strongly associated with LV hypertrophy than obesity. Among metabolic factors, SBP was found to show the strongest association with LVMI.

Overall, poor metabolic health status was associated with more adverse changes in LV structure and function than elevated BMI. Most parameters that differed significantly among the metabolic phenotypes worsened in the order of MHNW, MHOW, MHO and metabolic unhealthy groups, with metabolic derangement being more detrimental than higher BMI. Interestingly, obesity showed different significance when stratified by metabolic health status: MHO was associated with worse GLS, greater RWT and impaired diastolic function in the metabolically healthy stratum; however, within the metabolically unhealthy stratum, most of these LV parameters were not different among the BMI groups. This suggests that the effect of obesity may differ in magnitude according to presence of MetS. Adverse myocardial changes related to other metabolic factors could be more dominant in the metabolically unhealthy group; thus, obesity exerts less of an effect. Our findings also support that MHO subjects are not truly ‘healthy’ and show subclinical adverse changes in cardiac structure and function, and that MHO seems to be an intermediate phenotype between MHNW and metabolically unhealthy. This supports the assertion that obesity has adverse cardiovascular effects, regardless of metabolic disease.

### Study limitations

There are some limitations to our study. First, this is a retrospective cross-sectional study, so causal inferences cannot be drawn, and how these adverse myocardial changes in obesity and poor metabolic health status affect clinical outcome are not known. Further studies are needed to examine how these cardiac changes are related to cardiovascular disease and how modification of obesity or metabolic risk factors can affect outcome. Second, measures of obesity other than BMI were not available in our database. BMI is an incomplete representation of obesity, and further analysis including waist circumference or body fat percentage could have provided a deeper understanding of the relationship between obesity and myocardial function. Third, a limitation of the GLS technique is that good image quality is required for acquisition, which limits its use in subjects with poor echo windows. Also, there is significant inter-vendor variability for GLS values in the same subject. However, we used the same vendor (GE) to measure GLS in all patients, so inter-vendor variability is not a problem in our study. Fourth, data on socioeconomic status was not available for the study patients. Fifth, our study population was restricted to Korean people without overt heart disease, and generalization of our results to other populations may require caution.

## Conclusion

Obesity and poor metabolic health were associated subclinical changes in myocardial structure, systolic and diastolic function (poorer GLS, higher LVMI and E/e´), but not with LV ejection fraction in subjects without overt heart disease. Poor metabolic health status was related to more adverse changes in LV structure and function than obesity, and among risk factors of MetS, elevated SBP showed the strongest association with these subclinical LV alterations; thus, more emphasis should be put on blood pressure control in risk counseling. In the metabolically healthy stratum, obesity was associated with worse LV function, supporting that metabolically healthy obese are not truly healthy. Subclinical changes in LV structure and function related to obesity and metabolic health status should be considered in the management and risk stratification of subjects even with preserved LVEF.

## Supporting information

S1 Fig Study inclusion flow. (TIF)

S1 Table Clinical characteristics of the study population by metabolic health and obesity. (DOCX)

S2 Table Significant p-values of *post hoc* analyses for Table 2. (DOCX)

S3 Table Linear regression analysis for the association between metabolic phenotypes and echocardiography parameters. (DOCX)

S4 Table Impact of metabolic phenotypic groups on echocardiography parameters according to sex. (DOCX)

S5 Table Comparison of echocardiography parameters among metabolic phenotypes, when metabolically healthy is defined as having no risk factors. (DOCX)

S6 Table Comparison of echocardiography parameters among BMI categories, stratified by metabolic health. (DOCX)

S1 Dataset Dataset of the study population. (SAV)
