# Supplementary figures and images for: Subclinical alterations in left ventricular structure and function according to obesity and metabolic health status
Source: PLoS One. 2019 Sep 12;14(9):e0222118. doi: 10.1371/journal.pone.0222118 (PMC6742457; doi:10.1371/journal.pone.0222118)

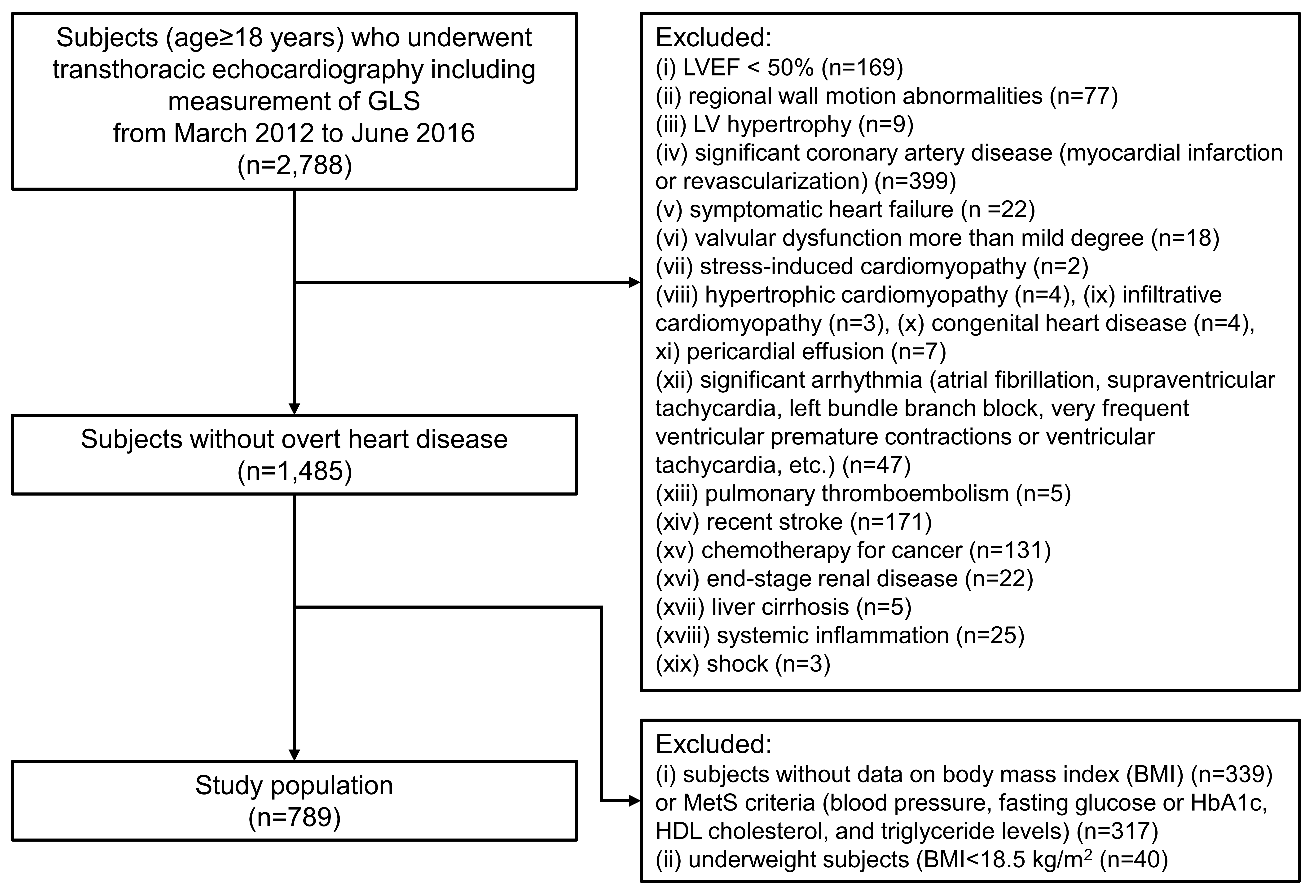

Supplement: S1 Fig — (TIF) [file pone.0222118.s001.tif]
