# Supplementary material for: Subclinical alterations in left ventricular structure and function according to obesity and metabolic health status
Source: PLoS One. 2019 Sep 12;14(9):e0222118. doi: 10.1371/journal.pone.0222118 (PMC6742457; doi:10.1371/journal.pone.0222118)
Supplement: S1 Table — (DOCX) [file pone.0222118.s002.docx]

**S1 Table. Clinical characteristics of the study population by metabolic health and obesity**

| **Characteristic** | **Metabolic health status** | | |  |  | **Obesity** | | | | |
| --- | --- | --- | --- | --- | --- | --- | --- | --- | --- | --- |
|  | **Healthy (n = 253)** | **Unhealthy (n = 536)** | ***P*** |  | **Normal weight (n = 251)** | | **Overweight (n = 206)** | **Obese (n = 332)** | ***P*** | ***P for trend*** |
| Age, years | 53.6±13.5 | 61.3±11.9 | < 0.001 |  | 59.6±13.9 | | 58.7±11.4 | 58.3±13.2 | 0.461 | 0.238 |
| Male sex, n (%) | 116 (45.8) | 284 (53.0) | 0.061 |  | 121 (48.2) | | 104 (50.5) | 175 (52.7) | 0.559 | 0.281 |
| Body mass index, kg/m^2^ | 23.7±2.7 | 25.0±2.9 | < 0.001 |  | 21.4±1.1 | | 24.1±0.6 | 27.3±2.1 | < 0.001 | < 0.001 |
| Systolic blood pressure, mmHg | 121±13 | 134 ±17 | < 0.001 |  | 128±18 | | 127±17 | 133±17 | 0.002 | 0.001 |
| Diastolic blood pressure, mmHg | 74.5±10.6 | 80.8±11.6 | < 0.001 |  | 77.3±11.9 | | 77.7±11.2 | 80.5±11.5 | 0.027 | 0.017 |
| Heart rate, beats/min | 65.7 ±9.9 | 68.5±11.5 | 0.010 |  | 67.4±10.9 | | 67.1±11.6 | 68.1±10.9 | 0.713 | 0.464 |
| Risk factors, n (%) |  |  |  |  |  | |  |  |  |  |
| Hypertension | 39 (15.4) | 369 (68.8) | < 0.001 |  | 109 (43.4) | | 91 (44.2) | 208 (62.7) | < 0.001 | < 0.001 |
| Diabetes mellitus | 10 (4.0) | 131 (24.4) | < 0.001 |  | 39 (15.5) | | 29 (14.1) | 73 (22.0) | 0.033 | 0.026 |
| Dyslipidemia | 10 (4.0) | 161 (30.0) | < 0.001 |  | 42 (16.7) | | 42 (20.4) | 87 (26.2) | 0.010 | 0.003 |
| Current smoker | 48 (19.0) | 96 (17.9) | 0.767 |  | 43 (17.1) | | 39 (18.9) | 62 (18.7) | 0.854 | 0.649 |
| Major laboratory results |  |  |  |  |  | |  |  |  |  |
| Fasting blood glucose, mg/dL | 98±16 | 120±62 | < 0.001 |  | 111±50 | | 110±28 | 116±65 | 0.344 | <0.001 |
| Hemoglobin A1c, % | 5.53±0.42 | 6.20±1.13 | < 0.001 |  | 5.88±0.93 | | 5.91±0.83 | 6.16±1.17 | 0.009 | <0.001 |
| Total cholesterol, mg/dL | 184±32 | 181±43 | 0.188 |  | 182±42 | | 183±38 | 181±40 | 0.768 | 0.595 |
| LDL cholesterol, mg/dL | 114±30 | 113±37 | 0.789 |  | 115±37 | | 113±33 | 113±35 | 0.845 | 0.755 |
| HDL cholesterol, mg/dL | 56.7±12.3 | 46.6±11.7 | < 0.001 |  | 53.0±14.2 | | 50.6±11.7 | 47.0±11.7 | < 0.001 | < 0.001 |
| Triglyceride, mg/dL | 92±33 | 138±103 | < 0.001 |  | 100±59 | | 124±73 | 141±115 | < 0.001 | < 0.001 |
| C-reactive protein, mg/dL | 0.34±1.21 | 0.43±1.35s | 0.483 |  | 0.46±1.77 | | 0.29±0.83 | 0.43±1.14 | 0.453 | < 0.001 |
| GFR, mL/min/1.73m^2^ | 94.1±21.7 | 87.1±22.6 | < 0.001 |  | 92.1±21.2 | | 86.5±19.1 | 88.9±25.3 | 0.033 | 0.010 |
| Medications, n (%) |  |  |  |  |  | |  |  |  |  |
| RAAS blocker | 26 (10.3) | 230 (42.9) | < 0.001 |  | 57 (22.7) | | 61 (29.6) | 138 (41.6) | < 0.001 | < 0.001 |
| Beta blocker | 29 (11.5) | 130 (24.3) | < 0.001 |  | 40 (15.9) | | 42 (20.4) | 77 (23.2) | 0.096 | 0.032 |
| Calcium channel blocker | 45 (17.8) | 226 (42.2) | < 0.001 |  | 75 (29.9) | | 64 (31.1) | 132 (39.8) | 0.023 | 0.010 |
| Diuretic | 8 (3.2) | 40 (7.5) | 0.018 |  | 13 (5.2) | | 11 (5.3) | 24 (7.2) | 0.517 | 0.290 |
| Statin | 61 (24.1) | 310 (57.8) | < 0.001 |  | 107 (42.6) | | 89 (43.2) | 175 (52.7) | 0.024 | 0.012 |

LDL, low-density lipoprotein; HDL, high-density lipoprotein; GFR, glomerular filtration rate; RAAS, renin-angiotensin-aldosterone system.
