# Supplementary material for: Subclinical alterations in left ventricular structure and function according to obesity and metabolic health status
Source: PLoS One. 2019 Sep 12;14(9):e0222118. doi: 10.1371/journal.pone.0222118 (PMC6742457; doi:10.1371/journal.pone.0222118)
Supplement: S2 Table — (DOCX) [file pone.0222118.s003.docx]

**S2 Table. Significant p-values of *post hoc* analyses for Table 2**

| **Echocardiography parameters** | **MHNW** | **MHOW** | **MHO** | **MUNW** | **MUOW** | **MUO** | ***P (among groups)*** |
| --- | --- | --- | --- | --- | --- | --- | --- |
| GLS, % | -19.91±2.29^a,b,c^ | -19.53±2.20^d^ | -19.05±2.19 | -18.84±2.80^a^ | -18.50±2.55^b^ | -18.48±2.40^c,d^ | < 0.001 |
| vs. MHNW | - | - | - | - | - | - |  |
| vs. MHOW |  | - | - | - | - | - |  |
| vs. MHO |  |  | - | - | - | - |  |
| vs. MUNW | 0.009 |  |  | - | - | - |  |
| vs. MUOW | <0.001 |  |  |  | - | - |  |
| vs. MUO | <0.001 | 0.015 |  |  |  | - |  |
| LVMI, g/m^2^ | 78.1±15.2^a,b,c^ | 78.1±15.3^d,e,f^ | 82.6±18.3^g^ | 88.9±19.6^a,d^ | 91.4±20.3^b,e,g^ | 86.0±17.3^c,f^ | < 0.001 |
| vs. MHNW | - | - | - | - | - | - |  |
| vs. MHOW |  | - | - | - | - | - |  |
| vs. MHO |  |  | - | - | - | - |  |
| vs. MUNW | <0.001 | <0.001 |  | - | - | - |  |
| vs. MUOW | <0.001 | <0.001 | 0.019 |  | - | - |  |
| vs. MUO | 0.002 | 0.011 |  |  |  | - |  |
| RWT | 0.34±0.04^a,b,c,d^ | 0.34±0.04^e,f,g^ | 0.36±0.05^a^ | 0.36±0.05^b,e^ | 0.38±0.04^c,f^ | 0.37±0.05^d,g^ | < 0.001 |
| vs. MHNW | - | - | - | - | - | - |  |
| vs. MHOW |  | - | - | - | - | - |  |
| vs. MHO | 0.029 |  | - | - | - | - |  |
| vs. MUNW | 0.003 | 0.014 |  | - | - | - |  |
| vs. MUOW | <0.001 | <0.001 |  |  | - | - |  |
| vs. MUO | <0.001 | 0.001 |  |  |  | - |  |
| A, m/s | 0.63±0.16^a,b,c^ | 0.65±0.17^d^ | 0.70±0.21 | 0.78±0.19^a^ | 0.78±0.19^b^ | 0.81±0.58^c,d^ | < 0.001 |
| vs. MHNW | - | - | - | - | - | - |  |
| vs. MHOW |  | - | - | - | - | - |  |
| vs. MHO |  |  | - | - | - | - |  |
| vs. MUNW | 0.011 |  |  | - | - | - |  |
| vs. MUOW | 0.028 |  |  |  | - | - |  |
| vs. MUO | <0.001 | 0.017 |  |  |  | - |  |
| E/A | 1.15±0.42^a,b,c^ | 1.08±0.37^d,e,f^ | 1.02±0.42 | 0.88±0.29^a,d^ | 0.86±0.28^b,e^ | 0.86±0.28^c,f^ | < 0.001 |
| vs. MHNW | - | - | - | - | - | - |  |
| vs. MHOW |  | - | - | - | - | - |  |
| vs. MHO |  |  | - | - | - | - |  |
| vs. MUNW | <0.001 | 0.002 |  | - | - | - |  |
| vs. MUOW | <0.001 | <0.001 |  |  | - | - |  |
| vs. MUO | <0.001 | <0.001 |  |  |  | - |  |
| e′, cm/s | 9.6±7.2^a,b,c^ | 8.0±2.6^d,e,f^ | 7.4±2.4 | 6.8±2.1^a,d^ | 6.8±2.0^b,e^ | 6.6±1.8^b,e^ | < 0.001 |
| vs. MHNW | - | - | - | - | - | - |  |
| vs. MHOW |  | - | - | - | - | - |  |
| vs. MHO |  |  | - | - | - | - |  |
| vs. MUNW | 0.003 | 0.024 |  | - | - | - |  |
| vs. MUOW | 0.002 | 0.015 |  |  | - | - |  |
| vs. MUO | 0.001 | 0.001 |  |  |  | - |  |
| E/e′ | 7.97±2.14^a,b,c,d^ | 8.83±2.55^e,f^ | 9.46±3.29^a^ | 10.20±3.38^b,e^ | 9.97±3.07^c^ | 10.15±3.18^d,f^ | < 0.001 |
| vs. MHNW | - | - | - | - | - | - |  |
| vs. MHOW |  | - | - | - | - | - |  |
| vs. MHO | 0.024 |  | - | - | - | - |  |
| vs. MUNW | <0.001 | 0.015 |  | - | - | - |  |
| vs. MUOW | <0.001 |  |  |  | - | - |  |
| vs. MUO | <0.001 | 0.004 |  |  |  | - |  |
| LAVI, mL/m^2^ | 26.6±6.6^a,b,c^ | 27.6±6.6 | 27.3±6.3 | 30.4±9.5^a^ | 30.5±11.0^b^ | 29.3±7.6^c^ | < 0.001 |
| vs. MHNW | - | - | - | - | - | - |  |
| vs. MHOW |  | - | - | - | - | - |  |
| vs. MHO |  |  | - | - | - | - |  |
| vs. MUNW | 0.004 |  |  | - | - | - |  |
| vs. MUOW | 0.013 |  |  |  | - | - |  |
| vs. MUO | 0.009 |  |  |  |  | - |  |

Superscript letters indicate significant differences between the marked groups in *post hoc* analysis. SPSS Bonferroni-adjusted p-values (raw p-value multiplied by the number of comparisons) are reported; only significant p-values (<0.05) are shown in the table.
