# Supplementary material for: Subclinical alterations in left ventricular structure and function according to obesity and metabolic health status
Source: PLoS One. 2019 Sep 12;14(9):e0222118. doi: 10.1371/journal.pone.0222118 (PMC6742457; doi:10.1371/journal.pone.0222118)
Supplement: S3 Table — (DOCX) [file pone.0222118.s004.docx]

**S3 Table. Linear regression analysis for the association between metabolic phenotypes and echocardiography parameters**

| **Parameter** | **Groups** | | | |
| --- | --- | --- | --- | --- |
|  | ***Unadjusted*** | | ***Adjusted^*^*** | |
|  | ***β*** | ***P*** | ***β*** | ***P*** |
| LVEF, % | 0.039 | 0.700 | 0.049 | 0.630 |
| GLS, % | 0.281 | **< 0.001** | 0.235 | **< 0.001** |
| LVMI, g/m^2^ | 1.902 | **< 0.001** | 1.389 | **< 0.001** |
| RWT | 0.006 | **< 0.001** | 0.005 | **< 0.001** |
| E, m/s | -0.004 | 0.435 | < 0.001 | 0.923 |
| A, m/s | 0.037 | **< 0.001** | 0.026 | **< 0.001** |
| E/A | -0.058 | **< 0.001** | -0.036 | **< 0.001** |
| DT, ms | 3.148 | **0.001** | 1.937 | **0.041** |
| e′, cm/s | -0.005 | **0.001** | -0.004 | **< 0.001** |
| E/e′ | 0.396 | **< 0.001** | 0.293 | **< 0.001** |
| LAVI, mL/m^2^ | 0.588 | **< 0.001** | 0.173 | **0.023** |
| TR Vmax, m/s | 0.018 | **0.006** | 0.011 | 0.099 |

Linear regression analysis done with groups as a continuous variable in the order of MHNW (0, reference), MHOW (1), MHO (2), MUNW (3), MUOW (4), and MUO (5). *β* estimate represents the change in each echocardiography parameter for 1-increase in group number.

*: adjusted for age and sex.

MHNW, metabolically healthy normal weight; MHOW, metabolically healthy overweight; MHO, metabolically healthy obese; MUNW, metabolically unhealthy normal weight; MUOW, metabolically unhealthy overweight; MUO, metabolically unhealthy obese; LVEF, left ventricular ejection fraction; GLS, global longitudinal strain; LVMI, left ventricular mass index; RWT, relative wall thickness; DT, deceleration time; LAVI, left atrial volume index; TR, tricuspid regurgitation; Vmax, maximal velocity.
