# Supplementary material for: Subclinical alterations in left ventricular structure and function according to obesity and metabolic health status
Source: PLoS One. 2019 Sep 12;14(9):e0222118. doi: 10.1371/journal.pone.0222118 (PMC6742457; doi:10.1371/journal.pone.0222118)
Supplement: S4 Table — (DOCX) [file pone.0222118.s005.docx]

**S4 Table. Impact of metabolic phenotypic groups on echocardiography parameters according to sex**

| **Echocardiography parameters** |  | **MHNW** | **MHOW** | **MHO** | **MUNW** | **MUOW** | **MUO** | ***P (group)*** | ***P (sex)*** | ***P for interaction (group*sex)*** |
| --- | --- | --- | --- | --- | --- | --- | --- | --- | --- | --- |
| LVEF, % | Men | 65.4 | 66.5 | 66.2 | 66.4 | 65.9 | 66.4 | 0.926 | **0.005** | 0.658 |
|  | Women | 67.8 | 66.2 | 67.2 | 67.6 | 67.2 | 67.5 |  |  |  |
| GLS, % | Men | -18.74 | -18.29 | -18.36 | -18.09 | -17.79 | -17.81 | **< 0.001** | **< 0.001** | 0.681 |
|  | Women | -20.83 | -20.54 | -19.73 | -19.62 | -19.33 | -19.26 |  |  |  |
| LVMI, g/m^2^ | Men | 81.4 | 82.0 | 84.0 | 91.7 | 93.3 | 85.7 | **< 0.001** | **0.003** | 0.363 |
|  | Women | 75.5 | 74.9 | 81.2 | 86.0 | 89.1 | 86.4 |  |  |  |
| RWT | Men | 0.351 | 0.356 | 0.370 | 0.371 | 0.381 | 0.371 | **< 0.001** | **< 0.001** | 0.813 |
|  | Women | 0.335 | 0.331 | 0.360 | 0.357 | 0.374 | 0.362 |  |  |  |
| E, m/s | Men | 0.610 | 0.597 | 0.652 | 0.643 | 0.621 | 0.613 | 0.954 | **< 0.001** | 0.500 |
|  | Women | 0.726 | 0.713 | 0.671 | 0.682 | 0.657 | 0.707 |  |  |  |
| A, m/s | Men | 0.597 | 0.616 | 0.693 | 0.744 | 0.725 | 0.712 | **< 0.001** | **0.002** | 0.203 |
|  | Women | 0.651 | 0.684 | 0.710 | 0.828 | 0.836 | 0.924 |  |  |  |
| E/A | Men | 1.12 | 1.06 | 1.01 | 0.88 | 0.88 | 0.91 | **< 0.001** | 0.696 | 0.193 |
|  | Women | 1.17 | 1.10 | 1.02 | 0.88 | 0.88 | 0.80 |  |  |  |
| DT, ms | Men | 210 | 201 | 214 | 209 | 215 | 213 | **0.023** | 0.835 | 0.527 |
|  | Women | 197 | 197 | 211 | 211 | 220 | 220 |  |  |  |
| e′, cm/s | Men | 10.2 | 8.1 | 7.2 | 7.1 | 7.2 | 6.9 | **< 0.001** | 0.058 | 0.682 |
|  | Women | 9.0 | 7.9 | 7.6 | 6.6 | 6.3 | 6.3 |  |  |  |
| E/e′ | Men | 7.31 | 7.91 | 9.37 | 9.45 | 9.08 | 9.12 | **< 0.001** | **< 0.001** | 0.258 |
|  | Women | 8.48 | 9.57 | 9.54 | 11.0 | 11.0 | 11.28 |  |  |  |
| LAVI, mL/m^2^ | Men | 25.9 | 27.7 | 26.6 | 29.8 | 28.4 | 28.0 | **< 0.001** | **0.005** | 0.322 |
|  | Women | 27.2 | 27.4 | 28.0 | 31.0 | 33.0 | 30.9 |  |  |  |
| TR Vmax, m/s | Men | 2.13 | 2.22 | 2.15 | 2.26 | 2.20 | 2.25 | 0.079 | **0.021** | 0.648 |
|  | Women | 2.22 | 2.20 | 2.29 | 2.27 | 2.27 | 2.31 |  |  |  |

Mean values of echocardiography parameters for each group are shown. *P* values are for comparison with two-way analysis of variance (ANOVA) to assess the effects of metabolic phenotype groups, sex, and the interaction between groups and sex.

MHNW, metabolically healthy normal weight; MHOW, metabolically healthy overweight; MHO, metabolically healthy obese; MUNW, metabolically unhealthy normal weight; MUOW, metabolically unhealthy overweight; MUO, metabolically unhealthy obese; LVEF, left ventricular ejection fraction; GLS, global longitudinal strain; LVMI, left ventricular mass index; RWT, relative wall thickness; DT, deceleration time; LAVI, left atrial volume index; TR, tricuspid regurgitation; Vmax, maximal velocity.
