# Supplementary material for: Subclinical alterations in left ventricular structure and function according to obesity and metabolic health status
Source: PLoS One. 2019 Sep 12;14(9):e0222118. doi: 10.1371/journal.pone.0222118 (PMC6742457; doi:10.1371/journal.pone.0222118)
Supplement: S5 Table — (DOCX) [file pone.0222118.s006.docx]

**S5 Table. Comparison of echocardiography parameters among metabolic phenotypes, when metabolically healthy is defined as having no risk factors**

| **Echocardiography parameters** | **MHNW (n = 33)** | **MHOW (n = 22)** | **MHO (n = 16)** | **MUNW (n = 218)** | **MUOW (n = 184)** | **MUO (n = 316)** | ***P*** | ***P for trend*** |
| --- | --- | --- | --- | --- | --- | --- | --- | --- |
| LVEF, % | 66.1 | 66.1 | 66.0 | 67.0 | 66.5 | 66.9 | 0.743 | 0.277 |
| GLS, % | -20.12 | -20.40 | -19.88 | -19.19 | -18.70 | -18.53 | **< 0.001** | **<0.001** |
| LVMI, g/m^2^ | 77 | 71.7 | 80.5 | 85.3 | 88.2 | 85.6 | **< 0.001** | **< 0.001** |
| RWT | 0.34 | 0.34 | 0.36 | 0.36 | 0.37 | 0.37 | **< 0.001** | **< 0.001** |
| E, m/s | 0.67 | 0.68 | 0.69 | 0.67 | 0.64 | 0.66 | 0.883 | 0.407 |
| A, m/s | 0.58 | 0.63 | 0.61 | 0.74 | 0.74 | 0.8 | **0.004** | **< 0.001** |
| E/A | 1.24 | 1.15 | 1.23 | 0.96 | 0.92 | 0.88 | **< 0.001** | **< 0.001** |
| DT, ms | 200 | 191 | 202 | 208 | 213 | 216 | **0.005** | **0.007** |
| e′, cm/s | 9.5 | 8.4 | 8.6 | 7.8 | 7.1 | 6.7 | **< 0.001** | **< 0.001** |
| E/e′ | 7.53 | 8.87 | 8.13 | 9.47 | 9.63 | 10.11 | **< 0.001** | **< 0.001** |
| LAVI, mL/m^2^ | 26 | 28.1 | 27.9 | 29.1 | 29.6 | 29 | 0.343 | **0.037** |
| TR Vmax, m/s | 2.1 | 2.3 | 2.2 | 2.3 | 2.2 | 2.3 | **0.016** | **0.041** |

Mean values of echocardiography parameters for each group are shown. *P* values are for comparison among groups with analysis of variance (ANOVA) or Welch test; bold values indicate significant differences of *P* < 0.05. P for trend was analyzed in the order of MHNW, MHOW, MHO, MUNW, MUOW and MUO.

MHNW, metabolically healthy normal weight; MHOW, metabolically healthy overweight; MHO, metabolically healthy obese; MUNW, metabolically unhealthy normal weight; MUOW, metabolically unhealthy overweight; MUO, metabolically unhealthy obese; LVEF, left ventricular ejection fraction; GLS, global longitudinal strain; LVMI, left ventricular mass index; RWT, relative wall thickness; DT, deceleration time; LAVI, left atrial volume index; TR, tricuspid regurgitation; Vmax, maximal velocity.
