# Supplementary material for: Subclinical alterations in left ventricular structure and function according to obesity and metabolic health status
Source: PLoS One. 2019 Sep 12;14(9):e0222118. doi: 10.1371/journal.pone.0222118 (PMC6742457; doi:10.1371/journal.pone.0222118)
Supplement: S6 Table — (DOCX) [file pone.0222118.s007.docx]

**S6 Table. Comparison of echocardiography parameters among BMI categories, stratified by metabolic health**

| **Parameter** | **Metabolically healthy** | | |  |  | **Metabolically unhealthy** | | |  |
| --- | --- | --- | --- | --- | --- | --- | --- | --- | --- |
|  | **MHNW (n = 111)** | **MHOW (n = 76)** | **MHO (n = 66)** | ***P^*^*** |  | **MUNW (n = 140)** | **MUOW (n = 130)** | **MUO (n = 266)** | ***P^*^*** |
| LVEF, % | 66.8±4.5 | 66.3±4.5 | 66.7±4.2 | 0.782 |  | 66.9±6.6 | 66.5±5.7 | 66.9±4.4 | 0.728 |
| GLS, % | -19.91±2.29^a^ | -19.53±2.20 | **-19.05±2.19^a^** | **0.048** |  | -18.84±2.80 | -18.50±2.55 | -18.48±2.40 | 0.385 |
| LVMI, g/m^2^ | 78.1±15.2 | 78.1±15.3 | 82.6±18.3 | 0.147 |  | 88.9±19.6 | 91.4±20.3^b^ | 86.0±17.3^b^ | **0.023** |
| RWT | 0.34±0.04 | 0.34±0.04 | **0.36±0.05^*^** | **0.002** |  | 0.36±0.05 | 0.38±0.04 | 0.37±0.05 | 0.051 |
| E, m/s | 0.68±0.16 | 0.66±0.16 | 0.66±0.19 | 0.815 |  | 0.66±0.16 | 0.64±0.14 | 0.66±0.35 | 0.726 |
| A, m/s | 0.63±0.16^a^ | 0.65±0.17 | **0.70±0.21^a^** | **0.027** |  | 0.78±0.19 | 0.78±0.19 | 0.81±0.58 | 0.720 |
| E/A | 1.15±0.42 | 1.08±0.37 | 1.02±0.42 | 0.097 |  | 0.88±0.29 | 0.86±0.28 | 0.86±0.28 | 0.818 |
| DT, ms | 203±43 | 199±42 | 212±46 | 0.181 |  | 210±53 | 217±44 | 216±50 | 0.374 |
| e′, cm/s | 9.6±7.2^a^ | 8.0±2.6 | **7.4±2.4^a^** | **0.017** |  | 6.8±2.1 | 6.8±2.0 | 6.6±1.8 | 0.506 |
| E/e′ | 7.97±2.14^*^ | **8.83±2.55** | **9.46±3.29** | **0.001** |  | 10.20±3.38 | 9.97±3.07 | 10.15±3.18 | 0.832 |
| LAVI, mL/m^2^ | 26.6±6.6 | 27.6±6.6 | 27.3±6.3 | 0.608 |  | 30.4±9.5 | 30.5±11.0 | 29.3±7.6 | 0.358 |
| TR Vmax, m/s | 2.2±0.3 | 2.2±0.3 | 2.2±0.2 | 0.645 |  | 2.3±0.3 | 2.2±0.3 | 2.3±0.2 | 0.359 |

Groups were stratified by metabolic health, and *P*-values are for comparisons among BMI groups within each strata using analysis of variance (ANOVA) or Welch test, with *post hoc* analysis. Bold values indicate significant differences of *P* < 0.05, and parameters that differ significantly in obese or overweight compared to normal weight subjects within each strata. Asterisks (*) indicate significant differences with the rest of the groups and superscript letters indicate significant differences between the marked groups in post hoc analysis.

BMI, body mass index; MHNW, metabolically healthy normal weight; MHOW, metabolically healthy overweight; MHO, metabolically healthy obese; MUNW, metabolically unhealthy normal weight; MUOW, metabolically unhealthy overweight; MUO, metabolically unhealthy obese; LVEF, left ventricular ejection fraction; GLS, global longitudinal strain; LVMI, left ventricular mass index; RWT, relative wall thickness; DT, deceleration time; LAVI, left atrial volume index; TR, tricuspid regurgitation; Vmax, maximal velocity.
